# Direct recognition of pathogen effectors by plant NLR immune receptors and downstream signalling

**DOI:** 10.1042/EBC20210072

**Published:** 2022-09-30

**Authors:** Jian Chen, Xiaoxiao Zhang, John P. Rathjen, Peter N. Dodds

**Affiliations:** 1 Commonwealth Scientific and Industrial Research Organization, Agriculture and Food, Canberra, ACT 2601, Australia; 2 Plant Sciences Division, Research School of Biology, The Australian National University, Canberra, ACT 2600, Australia

**Keywords:** effector, immune receptor, signalling

## Abstract

Plants deploy extracellular and intracellular immune receptors to sense and restrict pathogen attacks. Rapidly evolving pathogen effectors play crucial roles in suppressing plant immunity but are also monitored by intracellular nucleotide-binding, leucine-rich repeat immune receptors (NLRs), leading to effector-triggered immunity (ETI). Here, we review how NLRs recognize effectors with a focus on direct interactions and summarize recent research findings on the signalling functions of NLRs. Coiled-coil (CC)-type NLR proteins execute immune responses by oligomerizing to form membrane-penetrating ion channels after effector recognition. Some CC-NLRs function in sensor–helper networks with the sensor NLR triggering oligomerization of the helper NLR. Toll/interleukin-1 receptor (TIR)-type NLR proteins possess catalytic activities that are activated upon effector recognition-induced oligomerization. Small molecules produced by TIR activity are detected by additional signalling partners of the EDS1 lipase-like family (enhanced disease susceptibility 1), leading to activation of helper NLRs that trigger the defense response.

## Introduction

**Figure 1:**
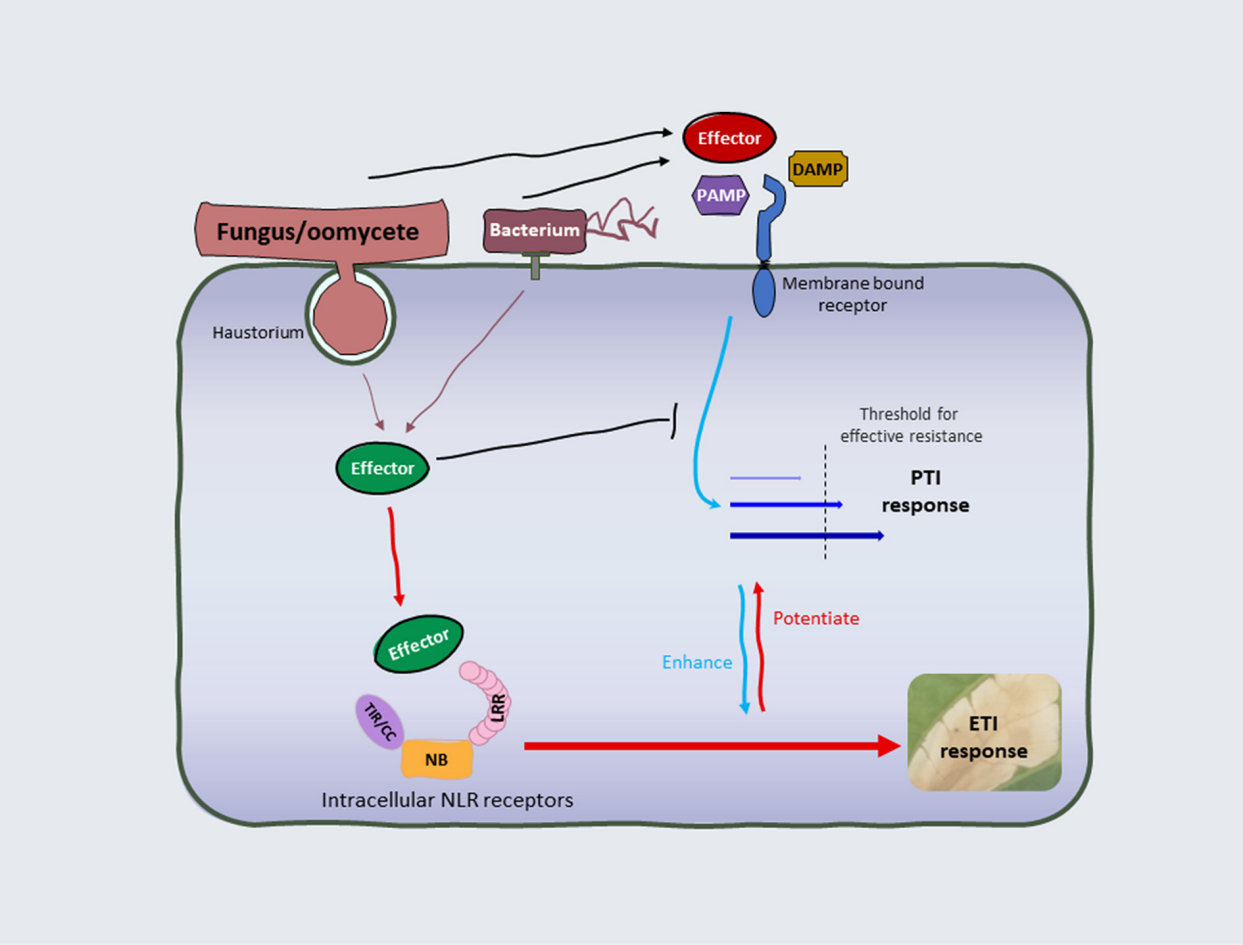
Overview of plant immunity In the extracellular space, PAMPs, DAMPs, or apoplastic effector proteins released from pathogens are recognized by cell surface membrane-bound receptors, inducing PTI. To suppress PTI, bacterial pathogens inject effectors into the host cell through a type-III secretion system, while fungi and oomycetes develop specialized structures such as haustoria to deliver effectors. Intracellular NLR receptors recognize specific effectors and trigger ETI, which is often associated with cell death at the infection sites. ETI can potentiate PTI by up-regulation of the underlying genes, while activation of PTI can enhance the defense response triggered by ETI. PTI and ETI work together to provide robust effective resistance against pathogens.

Plants and animals are continuously coresident with microbes, many of which are potential pathogens. While animals deploy specialized mobile cells within a circulatory system as the basis of adaptive immunity, plants rely on a cell-autonomous immune system to detect and restrict pathogen attacks. This comprises two main layers of recognition that operate either at the cell surface or in the host cytoplasm as illustrated in Figure 1 [1–3]. Pattern recognition receptors (PRRs) localized in the plasma membrane (PM) monitor the extracellular environment for the presence of pathogens through recognition of pathogen-associated molecular patterns (PAMPs), damage-associated molecular patterns (DAMPs), or apoplastic effector proteins, causing pattern-triggered immunity (PTI) [4–7]. Many adapted pathogens deliver effector proteins directly into plant cells to suppress defense responses including PTI [1,7]. As a countermeasure, plants have evolved a second layer of recognition involving intracellular nucleotide-binding/leucine-rich-repeat receptors (NLRs), which recognize effectors directly or indirectly, inducing effector-triggered immunity (ETI) [1,8]. Although description of the molecular biology of ETI is relatively recent, its existence was first synthesized in the ‘gene-for-gene model’ [9] describing the genetic interaction between host Resistance ‘*R*’ genes (i.e., NLRs) and pathogen Avirulence ‘*Avr*’ genes (recognized effectors). PTI provides a broad-spectrum resistance to a wide range of nonadapted pathogens and as such contributes to basal immunity [4,10,11]. ETI provides robust defense responses that are often associated with cell death termed the hypersensitive response (HR) at infection sites to inhibit pathogen proliferation [10,12,13]. There is also evidence for cross-talk between ETI and PTI pathways that can enhance immune responses [14,15]. In this review, we discuss activation of ETI with a focus on the direct recognition of pathogen effectors by plant NLRs and subsequent signalling events, based on the most recent research advances.

## NLRs and effector recognition

NLR proteins occur in plants, animals, and fungi and share a basic protein architecture consisting of a conserved central nucleotide-binding (NB) domain, a C-terminal LRR domain, and distinct N-terminal domains. Plant NLRs can be subdivided into two main groups according to their N-terminal domains: those containing an N-terminal TIR (Toll/interleukin-1 receptor and resistance) domain (TIR-NLR or TNL), and those with an N-terminal CC (coiled-coil) domain (CC-NLR or CNL). A subset of CC-NLRs have CC domains related to RPW8 (resistance to powdery mildew 8) and are known as CC_R_-NLRs or RNLs [16,17]. Generally, the N-terminal CC or TIR domains act as the signalling moieties and are often sufficient to activate downstream responses alone [18–22]. The central NB domain acts as a molecular switch determining the ‘on’ and ‘off’ signalling states of the NLRs by binding ADP or ATP, respectively [23–26]. In many cases, the LRR domain is responsible for the specificity of effector recognition [17,25–27], and congruently, this is often where the greatest polymorphism lies in *NLR* gene families. However, in other cases, additional noncanonical domains in some NLRs can mediate recognition as described below [25,26].

**Figure 2:**
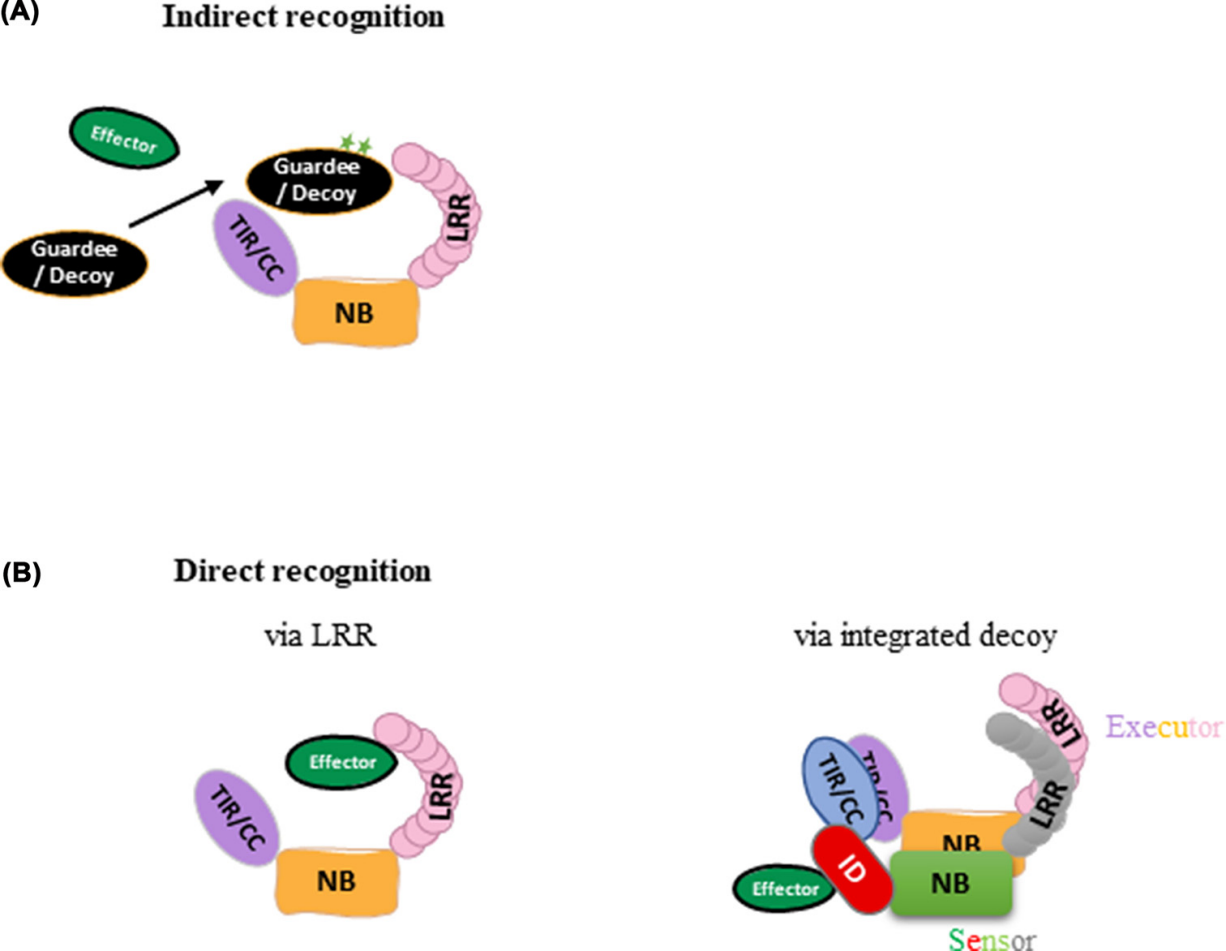
Models of effector recognition Plant NLR proteins recognize effectors (green) either directly or indirectly. (**A**) Indirect effector recognition occurs through monitoring effector-induced modifications of an intermediate guardee or decoy protein (black). (**B**) Direct effector recognition often occurs via interaction with the LRR domain (left) or with an ID (red), a decoy protein integrated with the NLR protein that mediates direct effector recognition. Such NLR-ID proteins often occur as part of a sensor/executor NLR pair, with both proteins required for specific resistance.

Recognition of effectors in the intracellular space of host cells is a central event in the activation of ETI. Plant NLR proteins can recognize effectors directly by physical interaction or indirectly through an intermediate partner as illustrated in Figure 2 [8,10,28]. Indirect effector recognition by NLRs involves recognition of changes induced in another host protein usually by the enzymatic activity of an effector [8]. Two conceptual models describe such indirect recognition (Figure 2A). In the ‘guard’ model, a host protein that is directly targeted by a pathogen effector as part of its virulence function is guarded by NLR proteins. In the ‘decoy’ model, a protein that mimics the effector target protein mediates recognition. NLR proteins monitor modifications of the ‘guard’ or ‘decoy’ proteins as indicators of pathogen infection that trigger their activation [29]. An advantage of indirect effector recognition for the host is that it is triggered by the function of a pathogen effector, which makes it difficult for the pathogen to escape recognition (i.e., by modifying or losing the effector) without losing a valuable virulence capability [28]. However, this mode of recognition generally requires that the pathogen effector has a protein-modifying activity. Direct recognition on the other hand involves detection of the presence of a pathogen effector by physical interaction with an NLR (Figure 2B), often mediated by the LRR domain [8]. Another mode of recognition is given by the integrated decoy model, which is a combination of direct recognition and the ‘decoy’ model. Here, NLR proteins contain additional integrated domains (ID) that mediate direct recognition by mimicking the protein targets of effectors [28,30–32]. Direct effector recognition has thus far been found more often for eukaryotic filamentous pathogens, whereas indirect recognition has been more often found for bacterial effectors [33,34]. Below we discuss examples of direct recognition between NLR proteins and effectors in more detail.

A number of examples of direct interactions are given in Table 1. One of the first examples to be characterized in detail was between variants of the flax NLR protein, L, and the flax rust fungus effector AvrL567. The specificity of recognition between L5, L6, and L7 and 12 AvrL567 variants is determined by polymorphic amino acids in the LRR domains of the NLR [27,35,36] as well as in surface-exposed residues of the AvrL567 protein [37]. This implies that a physical interface occurs between these regions of the two proteins that underlies recognition. Similarly, the recognition specificity of the barley NLR protein MLA is determined by polymorphisms in the LRR that are involved in physical interaction with AvrMla proteins from the barley powdery mildew pathogen [38]. A wheat homolog of MLA, Sr50, also directly recognizes its corresponding effector AvrSr50 from the wheat stem rust fungus [39]. Some AvrSr50 variants from virulent rust isolates escape Sr50 binding via one or a few mutations on the effector surface [40], while a central region of the LRR is the main contributor to its recognition specificity [41]. Likewise, recognition between the Arabidopsis NLR RPP1 and the downy mildew effector ATR1 involves interaction of effector surface residues with the C-terminal region of RPP1 including the LRR [42,43].

**Table 1:** Examples of direct receptor–effector recognitions

Resistance proteins	NLR type	Organism	Effector	Pathogen	Evidence	Domains involved in recognition	References
L5, L6, and L7	TIR	*Linum usitatissimum*	AvrL567	*Melampsora lini*	Y2H	LRR	[35,36]
M	TIR	*L. usitatissimum*	AvrM	*M. lini*	Y2H	Unknown	[103,104]
L2	TIR	*L. usitatissimum*	AvrL2	*M. lini*	Y2H	LRR	Unpublished data
MLA1, 7, 10, 13	CC	*Hordeum vulgare*	AVRa1, 7, 10, 13	*Blumeria graminis* f. sp. *hordei*	Y2H	LRR	[105,106]
Sr50	CC	*Secale cereale*	AvrSr50	*Puccinia graminis f. sp. tritici*	Y2H	LRR	[39,40]
Sr35	CC	*Triticum aestivum*	AvrSr35	*P. graminis f. sp. tritici*	CoIP, BiFC, structure	LRR	[107,108]
RppC	CC	*Zea mays* L.	AvrRppC	*P. polysora*	CoIP, BiFC	Unknown	[109]
RPP1	TIR-JID/PL	*Arabidopsis thaliana*	ATR1	*Hyaloperonospora arabidopsidis*	Structure	LRR and JID/PL	[26,110,111]
ROQ1	TIR-JID/PL	*N. benthamiana*	XopQ	*Xanthomonas*	Structure	LRR and JID/PL	[25,112]
N	TIR	*N. tabacum*	p50	*Tobacco mosaic virus*	Y2H	NB-LRR	[113]
Pi54	CC	*Oryza sativa*	AvrPi54	*Magnaporthe oryzae*	Y2H	unknown	[114]
Pi-ta	CC-ID	*O. sativa*	AvrPi-ta	*M. oryzae*	Y2H	LRR-ID	[115]
Sw-5b	CC	*Solanum lycopersicum*	Nsm	*Tomato-spotted wilt virus*	CoIP	LRR	[68]
RB/Rpi-blb1	CC	*Solanum bulbocastanum*	Avrblb1	*Phytophthora infestans*	Y2H	CC	[116]
Pik-1/Pik-2	CC-ID/CC	*O. sativa*	AvrPik	*M. oryzae*	Structure, Y2H	ID	[117,118]
RGA5/RGA4	CC-ID/CC	*O. sativa*	AvrPia, AVR1-CO39	*M. oryzae*	Y2H	ID	[50,119]
RRS1/RPS4	TIR-ID/TIR	*A. thaliana*	PopP2	*Ralstonia solanacearum*	Y2H	ID	[48,51,120–122]
RRS1/RPS4	TIR-ID/TIR	*A. thaliana*	AvrRps4	*Pseudomonas syringae*	Structure	ID	

Recent structural determination of the TIR-NLR proteins, ROQ1 and RPP1, in complex with their respective effectors, XopQ1 and ATR1, revealed key insights into the molecular basis of recognition [25,26,44]. In both cases, the LRR domains showed extensive physical contact with the effector proteins. However, in addition to this, a short domain located C-terminal to the LRR domains of ROQ1 and RPP1 called the C-JID/PL domain (jelly roll and Ig-like domain, or post-LRR) also contributed to the direct interaction with each effector. This was centered on a region of the JID/PL domain that shows high sequence variability. Mutagenesis assays confirmed that residues within the LRR and JID/PL domains determine the effector recognition specificity. The JID/PL domain exists in many but not all TIR-NLR proteins across multiple plant species [45,46], and could be a common effector recognition component of TNLs in addition to LRR domains.

NLRs with integrated decoy domains often occur as one member of an NLR pair, in which one protein known as the sensor NLR contains an ID that interacts directly with effectors to induce activation of the alternate executor (or helper) NLR (Figure 2B) [17,28,30,47]. Arabidopsis RRS1/RPS4 [48], rice Pik-1/Pik-2 [49], and RGA5/RGA4 pairs [50] are three well-known examples of paired sensor/executor NLRs. The sensor proteins Pik-1 and RGA5 of rice recognize different effectors from the rice blast fungus that bind to their HMA (heavy-metal associated) IDs and trigger immunity via the executors Pik-2 and RGA4, respectively [49,50]. The RRS1 sensor protein carries a WRKY domain as an ID and recognizes the unrelated bacterial effectors *Pseudomonas syringae* AvrRps4 and *Ralstonia solanacearum* PopP2, leading to immune responses mediated by the executor protein RPS4 [48,51]. These effectors normally target host WRKY transcription factors, but their action on the RRS1 WRKY ID leads to effector recognition. In the case of AvrRps4, physical interaction of the effector with the RRS1 WRKY domain disrupts an intramolecular interaction with another C-terminal domain thereby destabilizing the inactive state of the protein complex [52]. Overexpression of the executor NLRs, RPS4 and RGA4, causes autoactive cell death *in planta*, but overexpression of the sensor NLRs, RRS1 and RGA5, does not [53,54]. Moreover, the autoactivity of these executor NLRs can be suppressed by coexpression of RRS1 or RGA5. Hence, sensor NLRs can act as suppressors in some cases to inhibit executor NLRs in the absence of effector recognition. However, in other cases, paired NLRs work co-operatively. For instance, the rice CC-NLR pair Pikp-1/Pikp-2 requires both NLRs to trigger cell death, and neither of them is autoactive when expressed alone [55].

## NLR resistosomes and immune signalling

**Figure 3:**
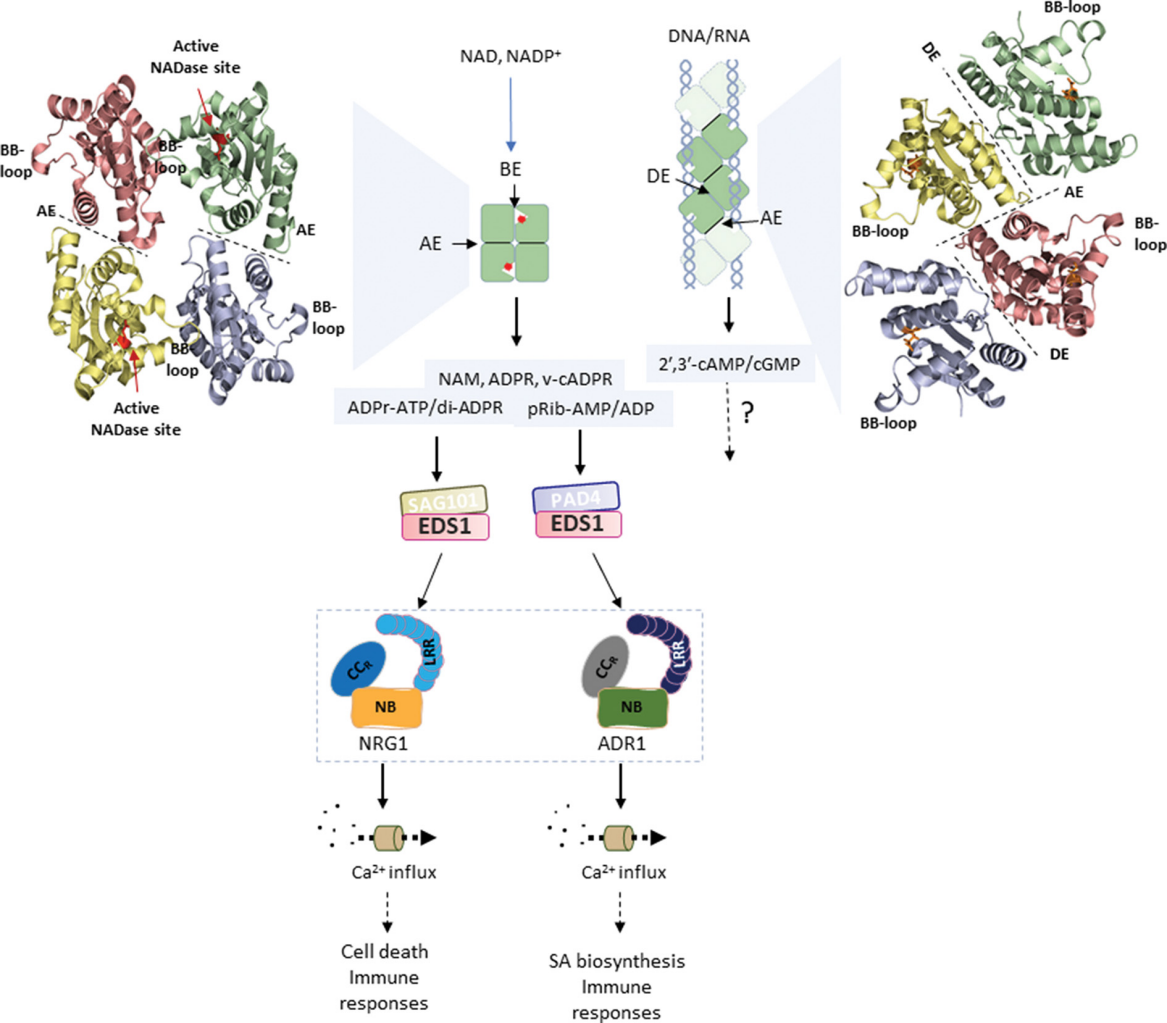
Working model for TIR activation and signalling pathways Upon TIR-NLR activation, resistosome formation causes the TIR domains (indicated by the green squares in the schematic model) to oligomerize through AE and BE interfaces to activate NADase activity, which cleaves NAD or NADP^+^ to generate various small molecules and ADP-ribosylation products (left). Alternatively, TIR domains may also self-associate through AE and DE interfaces to form helical filaments, which can bind and hydrolyze DNA/RNA and generate 2′,3′-cAMP/cGMP (right). Extreme left: Roq1TIR tetramer cryo-EM structure (PDB: 7JLX). Extreme right: L6TIR (PDB: 3OZI) tetramer structural model assembled through AE and DE interfaces. The TIR monomer subunit structures are shown in green, yellow, lavender, and salmon with the catalytic glutamate residues shown in red in the resistosome tetramer (left) and in orange in the AE–DE filament (right). The NADase activity products ADPr-ATP/di-ADPR bind to EDS1/SAG101 complexes, while pRibAMP/ADP binds to EDS1/PAD4 complexes. This results in activation of their helper CC_R_-NLRs, NRG1, and ADR1, respectively. NRG1s promote cell death, whereas ADR1s induce SA biosynthesis, achieved by triggering Ca^2+^ influx or through some other unknown mechanism(s). Solid arrows indicate confirmed links, dotted arrows indicate tentative pathways.

Several early studies showed that self-association of the TIR or CC signalling domains is a precondition of cell death activity. For example, mutations that disrupt the self-association of Sr33 or MLA10 CC domains abolish the cell death caused by either expression of the CC domain alone or the full-length CNL protein [21,56]. Similarly, the crystal structures of isolated plant TIR domains contain separate self-interaction interfaces, AE and DE (Figure 3), and mutational analyses have shown that both are necessary for TIR signalling [57–59]. These observations suggested that NLR proteins function via assembly into multimeric signalling complexes after effector detection [60]. In agreement with this, there is now direct structural evidence that three different NLRs self-associate to form higher-order ‘resistosome’ protein complexes after effector recognition [25,26,61], discussed in further detail below.

## CC-NLR activation and immune signalling

Structural determination of complexes containing the full-length CC-NLR protein ZAR1 revealed major conformational changes during its activation. This work identified three structural phases: an inactive monomeric state, an intermediate preactivation monomeric state, and the active state, which forms a wheel-like pentameric resistosome complex [24,61]. Inactive ZAR1 binds ADP, while an RKS1 pseudokinase molecule interacts with the LRR domain. This prerecognition complex detects the *Xanthomonas* effector AvrAC indirectly via a decoy protein kinase, PBS1-like protein 2 (PBL2). After uridylation by AvrAC, PBL2^UMP^ interacts with the ZAR1-RKS1 complex through RKS1, causing outwards rotation of the ZAR1 NB domain, which promotes ADP release and subsequent ATP binding. ATP binding induces further structural changes that assist formation of the active pentameric structure. The N-terminal α1 helix of the CC domain is exposed upon ZAR1 activation and in the resistosome complex, the five CC domains form a funnel-shaped structure with the α1 helices at the apex. The active ZAR1 resistosome associates with the PM and can act as a Ca^2+^ influx channel [61–64]. Mutation of negatively charged residues within the funnel-shaped CC domain structure abolish ion transport and cell death activity, suggesting that this structure inserts into the membrane to form a Ca^2+^ permeable channel that is necessary for immunity activation. Newly published research shows that the isolated CC domain of the Arabidopsis helper CC_R_-NLR NRG1.1 also adopts a four-helical bundle structure that closely resembles those of other plant CC domains and the inactive state of ZAR1 [18]. Moreover, the active *At*NRG1.1 protein oligomerizes and forms puncta in the PM, and both activated *At*NRG1.1 and *At*ADR1 can cause Ca^2+^ influx. Both channel activity and cell death induction also require conserved negatively charged residues in the N-terminal of the CC domains, as observed for ZAR1. This suggests that the CC_R_ domains of these proteins function similarly to the CC domains of other CNL proteins. Moreover, transcriptome analysis revealed that similar gene expression changes are triggered by activation of both CC_R_-NLRs and other CNLs [18,65]. Hence, formation of Ca^2+^ permeable channels might be a common mechanism for immune activation by CC-NLRs. There are, however, exceptions to this model. For instance, many of the CC-NLRs in the Solanaceae family operate within a sensor-helper network, where a variety of sensor CC-NLRs are involved in pathogen detection but require helper CC-NLRs of the NRC family to induce immune responses [66,67]. For instance, Sw-5b (Table 1) acts as a sensor that directly interacts with the Nsm protein of tomato-spotted wilt virus and requires NRC family helper NLRs for signalling [67,68]. A recent preprint [69] suggests that pathogen recognition by sensor CC-NLRs in this family leads to oligomerization and membrane association of the NRC helpers.

## TIR-NLR activation and immune signalling

TIR-NLRs adopt a very different signalling strategy compared with CC-NLRs. TIR domains possess NADase catalytic activity; upon self-association, TIRs hydrolyze NAD^+^ and generate a variant-cyclic-ADP-ribose products (v-cADPR), which is hypothesized to activate downstream signalling [19,20]. The TIR domain of the animal SARM1 protein and some bacterial and archaeal TIRs were first shown to have intrinsic NAD^+^ hydrolase activity [70,71]. Plant TIRs share similar structures to that of SARM1 TIR and possess NADase activity dependent on a conserved glutamate residue, *albeit* at much lower levels than SARM1 [19,20]. The NADase activity of isolated plant TIR domains requires self-association, with mutations in the AE or DE interfaces abolishing the NADase activity (as well as cell death signalling), while the activity can be enhanced by using molecular-crowding agents (e.g., polyethylene glycol) that promote self-association by increasing the effective protein concentration [19,20]. These observations suggest that plant TIRs need to form higher order structures for NADase activation.

The TIR-NLR proteins ROQ1 from *Nicotiana benthamiana* and RPP1 from Arabidopsis recognize their cognate effectors directly [25,26]. Unlike the ZAR1 resistosome that forms a disc-like pentamer structure with the N-terminal CC domain helix protruding from its center, ROQ1 and RPP1 form tetrameric structures more like a triple-layered cylinder. Four LRR-JID/PL domain-effector complexes spread out at the one end, while the NB domains oligomerize in the center and thereby drive the TIR domains to self-associate at the other end of the cylinder. TIR domain self-association occurs through two AE and BE interfaces as two asymmetric pairs (Figure 3). While the AE interface is conserved between the resistosome and isolated TIR domain structures, the BE interface is unique to the resistosome and was not observed in TIR X-ray structures, although it does involve some of the same residues involved in the DE interface. Formation of the ROQ1 and RPP1 tetramers exposes two NADase catalytic sites in the two BE interfaces, such that the tetramer includes two NADase-active TIR domains and two inactive domains. Fusion of plant TIRs to the multimerization domain of SARM1 (octamer-forming) or to NLRC4 (which contains about ten protomers in an open-ended ring) can activate cell death signalling in *N. benthamiana* [19,72]. Thus, TIR oligomerization works upstream of activation of NADase activity, and the stoichiometry of the activated TIR complex is not strictly limited to tetrameric structures. The presence of the BE interface in these resistosomes in place of the DE interface in some TIR-alone crystal structures suggested the possibility that the latter could be a crystal artefact. However, a recent study showed that the plant L7 TIR domain forms helical filaments with AE and DE interfaces that bind to dsDNA or dsRNA molecules within the helical groove and can hydrolyze these molecules to release 2′,3′-cAMP/cGMP [73]. This activity depends on the same active site residues involved in NADase activity. Hence, plant TIRs may adopt different interface combinations (AE+DE or AE+BE; Figure 3) to initiate either 2′,3′-cAMP/cGMP synthetase or NADase activity, respectively, which could both contribute to generation of downstream signalling molecules, possibly with different signalling outcomes [73].

## Signalling convergence and distribution: EDS1 and helper NLRs

Two levels of signalling components are required downstream of the TIR domain catalytic activity (NADase/2′,3′-cAMP/cGMP synthetase) leading to resistance outputs (Figure 3). The first level consists of members of the EDS1 family of lipase-like proteins. EDS1 forms mutually exclusive heterodimers with its family members PAD4 (phytoalexin deficient) or SAG101 (senescence-associated gene) [74–77]. The second level is composed of the helper CC_R_-NLRs, NRG1 and ADR1, that work co-operatively with either the EDS1-SAG101 or EDS1-PAD4 heterodimers, respectively, to mediate immunity [16,78–82]. It remains unclear how immune signals are transferred from TIRs to these downstream signalling partners.

EDS1 family proteins feature an N-terminal lipase-like α/β-hydrolase-fold domain and a C-terminal EP domain (named from the EDS1-PAD4 interaction), which is composed primarily of α-helical bundles [77]. Both EDS1 and PAD4 contain a conserved SDH catalytic triad in the lipase-like domain, and the EP domains have a conserved EPLDIA motif with unknown function [77,83–86]. The solved Arabidopsis EDS1-SAG101 heterodimer structure and the modelled EDS1-PAD4 complex show that the two heterodimers adopt similar interaction profiles [77]. Their interaction is mainly mediated by the N-terminal domains, with the αH helix in the EDS1 N-terminal lipase-like domain fitting neatly into the groove of the N-terminal domain of PAD4 or SAG101. The C-terminal EP domains interact weakly, creating cavity surfaces that are necessary for immune signalling mediated by both EDS1 heterodimers [77,79,83,84,86–88]. One hypothesis has been that the products of TIR catalytic activity bind to this cavity to modify EDS1 heterodimers, and two recent preprints confirm this hypothesis. Huang et al. [89] show that TIR catalytic activity results in the production of phosphoribosyl-AMP/ADP, which binds to the EDS1/PAD4 complex, while Jia et al. [90] show that TIR activity also leads to ADPr-ATP production and binding to the EDS1/SAG101 complex. In both cases, small-molecule binding causes conformational changes in the EDS1 complexes and results in engagement with the ADR1 or NRG1 helper NLRs, respectively.

The helper CC_R_-NLRs, ADR1 and NRG1, represent an ancient branch of the *CC-NLR* gene family that occurs in widely diverged plant lineages. This family is usually represented by only one or a few copies within each species in contrast with the broad diversity of other CNL and TNL families. CC_R_-NLRs from various species, especially Arabidopsis, are required for the function of all tested TNLs and some CNLs [16,65,91–94]. Studies in Arabidopsis revealed that ADR1 and NRG1 function differently: generally, *At*ADR1s are used by both TNLs and CNLs and function upstream of the SA pathway to restrict pathogen growth, whereas *At*NRG1s function mainly for cell death induction by TNLs. *At*ADR1s and *At*NRG1s work synergistically to provide effective resistance [78–81]. However, another study showed that ADR1 can also mediate cell death induced by RRS1/RPS4 in Arabidopsis when NRG1s and the SA pathway are blocked [95]. Transient overexpression of *Solanum tuberosum* ADR1 (*St*ADR1) or *Nb*NRG1 induces a resistance response that suppresses the accumulation of *potato virus X* without visible HR [16,91]. Thus, it is reasonable to conclude that both ADR1 and NRG1 possess transcriptional reprogramming and cell death-inducing capacities [95]. EDS1-PAD4 and EDS1-SAG101 heterodimers form two parallel signalling pathways with ADR1 and NRG1 acting downstream, respectively [79,81,86,87]. Several studies have suggested that the EDS1-SAG101 heterodimer works together with NRG1 in inducing cell death triggered by TIR-NLRs, whereas the EDS1-PAD4 heterodimer acting with ADR1 provides broader transcriptional defense in basal immunity used by both TNLs and CNLs [79,86,88,96,97]. All TIR-NLRs tested so far in *N. benthamiana* require EDS1-SAG101 and NRG1 for activation of immunity [79,80,87], while the *Nb*EDS1–*Nb*PAD4–*Nb*ADR1 module has not yet been shown to contribute to immunity in *N. benthamiana*. An expression profiling study showed that *Nb*NRG1 is required for 80% of the transcriptional changes induced by recognition of the bacterial XopQ1 effector in *N. benthamiana*, suggesting a major role in this response [80]. The remaining 20% of transcriptional changes may be controlled by other signalling proteins, possibly including *Nb*ADR1. The entire Arabidopsis *At*EDS1–*At*SAG101–*At*NRG1 module can be transferred into *N. benthamiana* to mediate TIR-NLR-triggered immunity [79]. However, combinations of these genes derived from different species (e.g., *At*EDS1 plus *Nb*SAG101), or the *At*EDS1–*At*PAD4–*At*ADR1 module, cannot confer cell death mediated by TIR-NLRs or restriction of pathogen growth in *N. benthamiana* [79,86,91]. This supports the idea that EDS1 family proteins and helper CC_R_-NLRs coevolved separately in different species.

In summary, EDS1 family proteins seem to act as a signalling convergence node where the EDS1-PAD4 and EDS1-SAG101 heterocomplexes work complementarily to collect immune signals from NLRs. These heterocomplexes further activate either ADR1 or NRG1 to execute different immune responses, which require their CC_R_ domains and Ca^2+^ channel activity. A reasonable hypothesis is that the EDS1 family node evolved as an adapter complex to connect TNLs to a pre-existing CNL signalling pathway. No TNL, SAG101, or NRG1 proteins exist in monocots, but the monocot TIR-only protein BdTIR possesses NADase activity and can induce EDS1- and NRG1-dependent cell death in *N. benthamiana* [16,20,79,83]. It would therefore be interesting to learn how TIR-only proteins function in monocot plants. Little is known of the physical connections between NLRs and EDS1. Some *Arabidopsis* TNLs including RPS4, RPS6, and SNC1 have been reported to interact with EDS1 [53,98–100]. Effector-dependent associations between EDS1-SAG101 and NRG1, and EDS1-PAD4 and ADR1, have been proposed in Arabidopsis based on genetic and molecular evidence [95]. However, how immune signalling is transferred from NLRs to the EDS1 node and then activates helper RNLs remains unclear. Apart from mediating ETI triggered by plant intracellular NLRs, the EDS1-PAD4-ADR1 module is reported to participate in immune responses mediated by the cell surface receptor RLP23, suggesting that it may also function as a convergence point for PTI and ETI [101]. Two recent studies in Arabidopsis reported that PTI and ETI mutually reinforce each other during resistance (Figure 1) [14,15]. The authors argue that PTI provides the primary defense against most pathogens, and that ETI mediated by intracellular NLR proteins enhances the transcription and protein stability of PRR signalling components, reversing the attenuation of PTI caused by pathogens. Conversely, ETI responses could be strongly enhanced by activation of plant cell surface immune receptors [102].

## Summary

- Plant NLRs have evolved direct and indirect recognition mechanisms to detect pathogens. Direct recognition is the dominant form in resistance to biotrophic filamentous pathogens such as fungi and oomycetes.
- Selection imposed by direct recognition leads to evolution of effector proteins through modification of surface residues that prevent interaction with host receptors.
- CNL and TNL proteins assemble into higher-order resistosomes after effector recognition. In CNL resistosomes, the CC domains seem to act as Ca^2+^ channels on the PM while oligomerization of TNLs activates TIR domain enzymatic functions that produce potential signalling molecules.
- EDS1–PAD4–ADR1 and EDS1–SAG101–NRG1 modules work differently and synergistically for ETI.
- Mapping physical connections between TIR-NLRs, EDS1 family proteins and NRG1 or ADR1, are required to understand immune signal transmission during ETI.

## Abbreviations

CC: coiled-coil
DAMP: damage-associated molecular pattern
EDS1: enhanced disease susceptibility 1
EP: EDS1-PAD4
ETI: effector-triggered immunity
HMA: heavy-metal associated
HR: hypersensitive response
ID: integrated domain
NB: nucleotide-binding
NLR: nucleotide-binding, leucine-rich repeat immune receptor
PAD4: phytoalexin deficient
PAMP: pathogen-associated molecular pattern
PBL2: PBS1-like protein 2
PRR: Pattern recognition receptor
PTI: pattern-triggered immunity
RPW8: resistance to powdery mildew 8
SAG101: senescence-associated gene
StADR1: *Solanum tuberosum* ADR1
TIR: Toll/interleukin-1 receptor
v-cADPR: variant-cyclic-ADP-ribose
